# Development of a sustainable access to medicine model in the Caribbean: a case study of the chronic disease assistance program

**DOI:** 10.1186/2052-3211-7-S1-P8

**Published:** 2014-12-17

**Authors:** Sandeep Maharaj, Sureshwar Pandey, Yashwant Pathak, Manthan Janodia

**Affiliations:** 1 The University of the West Indies, School of Pharmacy, St. Augustine, Trinidad; 2 University of South Florida, College of Pharmacy, Tampa, Florida, USA; 3 College of Pharmaceutical Sciences, Manipal University, Manipal, India

## Background

The Caribbean region is one where there is a marked increase in non-communicable diseases and at the same time there are significant financial constraints. This study seeks to develop a methodology for a sustainable supply chain mechanism for medicines which can be implemented in these countries. To develop this, the chronic disease assistance programme currently implemented in Trinidad and Tobago was assessed and a template was developed around this.

## Method

Data was collected via both Primary and Secondary sources. Primary data was collected via a structured questionnaire as well laboratory test on the quality of drugs found in the Supply Chain. Secondary data was taken from country reports, scholarly journal published articles and trade articles.

## Results

It was found that in the Trinidad and Tobago case, the engagement of the private sector has significantly reduced the patient waiting time in the hospital. It has assisted with the human resource deficiency in the public sector. However, there are significant systemic accountability gaps which need to be rectified in both the short term and long term to ensure a proper working system. The quality of medication in the parallel system was found to be of a good quality.

## Discussion

It was found in this study that in addition to financial limitations, there are other issues that require addressing such as bottlenecks in drug procurement and supply; lack of trained manpower; lack of co-ordination between various ministries and departments implementing the program as well as the inefficient use of technology in the appropriate implementation of the program. We have made suggestions for resolving these issues and if implemented would lead to creating a robust, sustainable transparent supply chain in the Caribbean.

## Lessons learned

There are numerous components to drug supply chain management in the Caribbean. However one needs to be very innovative in a financially, human resource and technology strained environment.

